# From quantum semantic states to quantum semantic dynamics: a multiscale theory of literary meaning evolution

**DOI:** 10.3389/fpsyg.2026.1948062

**Published:** 2026-10-28

**Authors:** Tzonelih Hwang

**Affiliations:** Independent Researcher, Tainan, Taiwan

**Keywords:** literary meaning, model comparison, quantum cognition, quantum semantics, question order

## Abstract

Literary interpretation changes as readers encounter new passages, answer interpretive questions, and revisit earlier material in light of later context. Quantum cognition provides a probability calculus for judgments whose probabilities depend on context and probe order, but the use of Hilbert-space notation alone does not establish nonclassical behavior. This article develops Quantum Semantic Dynamics (QSD) as a constrained quantum-probabilistic model and distinguishes three claim levels: quantum-inspired representation, quantum-probabilistic structure, and intrinsic nonclassicality. The present paper advances only the second. The confirmatory implementation restricts the local semantic state to a two-dimensional space, fixes probe geometry in an independent pilot sample, reconstructs a population-level 2 × 2 density state from three linearly independent binary probe bases, and gives the off-diagonal state components an operational meaning through cross-basis judgments. Contextual evolution is restricted to a one-parameter completely positive trace-preserving contraction toward a preregistered context-target state rather than an arbitrary latent transition. Two probes form the randomized A → B/B → A sequence used for the quantum question-order (QQ) diagnostic; the third is measured in an independent arm for state identification. The paper derives the QQ equality, states its assumptions, and adds an uncertainty-style consistency constraint for the frozen noncommuting probe geometry. QSD is compared with explicitly defined classical comparator classes ranging from a static commutative model to matched sequential latent-state models. Compatibility with QQ or the Hilbert representation is not treated as proof of intrinsic nonclassicality; such a claim would require a separate contextuality analysis against an explicitly defined classical representability criterion. The resulting architecture makes the mathematical status, empirical burden, and rejection conditions of QSD explicit before data collection.

## 1 Introduction

Literary meaning is temporally organized. A phrase that appears descriptive on first encounter can acquire relational, ethical, or existential force after a later scene; an apparently settled interpretation can reopen; and rereading can make an earlier detail function differently within the work as a whole. These effects are familiar to literary theory, where reading has long been treated as an interaction among textual structure, expectation, and retrospective integration (Iser, 1978; Ricoeur, 1984). They are also compatible with cognitive theories in which discourse comprehension depends on incremental construction, memory, and situation-model updating (Kintsch, 1988; Zwaan and Radvansky, 1998).

Quantum cognition offers a more specific modeling framework. Standard classical probability represents uncertain alternatives within a single event structure, while classical sequential models can additionally represent history-dependent updating. Quantum probability supplies a candidate calculus when the act or order of probing a judgment changes the state used for the next judgment and a shared noncommuting probe geometry is empirically useful. This approach has been used to model question-order effects, contextual judgment, and concept combination (Pothos and Busemeyer, 2009, 2013; Busemeyer and Bruza, 2012; Wang and Busemeyer, 2013). Quantum-inspired semantic research has likewise used vector and density-operator representations for ambiguity and compositional meaning (Piedeleu et al., 2015; Zhang et al., 2018), while empirical studies have examined quantum-like structure in text perception and metaphor comprehension (Surov et al., 2021; Fuyama, 2023). The usefulness of that representation remains an empirical question rather than a presumption of intrinsic nonclassicality.

Yin (2026) brought this family of ideas directly into dynamic literary interpretation, using The Old Man and the Sea to illustrate how a literary object can support context-sensitive semantic alternatives. The present paper takes the next step by isolating the smallest formal structure that makes a discriminating prediction about interpretive change and by comparing it with classical sequential models that already handle learning, memory, and context.

QSD is developed around four linked empirical requirements: identification of a common semantic state across interpretive bases; compatibility of focal probe sequences with the parameter-free QQ equality; representation of semantic reopening after later narrative information; and predictive gain from higher-scale context beyond matched classical sequential models. The first requirement concerns state identification, the second provides a model-specific structural test, and the latter two concern literary dynamics.

The paper is organized accordingly. Related work and terminology are consolidated first so that the quantum and classical claims are visible before the model is introduced. The model section then fixes a two-dimensional local semantic subspace, specifies how its inner-product geometry is calibrated independently of confirmatory responses, reconstructs the population state from three probe bases, and restricts contextual dynamics to a completely positive trace-preserving channel. The sequential section derives the QQ equality and an operational uncertainty constraint for the A/B probe pair. The final sections define classical comparator classes, preregisterable adjudication rules, and the boundary between support for a quantum-probabilistic model and evidence for intrinsic nonclassicality.

## 2 Related work and model positioning

### 2.1 Quantum cognition and quantum semantic modeling

Quantum cognition offers a Hilbert-space probability framework for judgments in which context and measurement order matter (Pothos and Busemeyer, 2009, 2013; Busemeyer and Bruza, 2012). For sequential binary judgments, Wang and Busemeyer (2013) derived the quantum question-order (QQ) equality: a parameter-free relation among the four joint outcomes observed under A → B and B → A order. The equality is stronger than the mere existence of an order effect because some standard Bayesian and Markov formulations do not generally satisfy it; however, sufficiently flexible classical contextual or sequential constructions may reproduce response tables that are also compatible with QQ. QSD therefore uses the equality as a model-specific diagnostic, not as a general witness of nonclassicality.

Quantum-inspired semantic modeling predates the present literary proposal. Open-system and categorical approaches have represented linguistic meaning with density operators and related transformations (Piedeleu et al., 2015), end-to-end quantum-like language models have used complex-valued state-measurement pipelines for question answering (Zhang et al., 2018), and text-perception studies have examined quantum-semantic structure in human judgments (Surov et al., 2021). Fuyama (2023) tested quantum-cognitive predictions in metaphor comprehension. Yin (2026) brought the approach directly into dynamic literary interpretation, using The Old Man and the Sea to illustrate context-sensitive semantic alternatives. QSD builds on this line but adds an explicit identification requirement: the same latent state must be reconstructable from a preregistered set of probes before its sequential behavior is used to test a quantum question-order constraint.

### 2.2 Quantum–classical architectures and classical alternatives

Recent hybrid architectures also sharpen the methodological standard for a quantum-inspired model. Zhang et al. (2025) developed a hierarchical entanglement-embedding network that makes its multi-grained semantic modules explicit and evaluates them against strong baselines. Yan et al. (2026) modeled multimodal sentiment and sarcasm with density-matrix evolution under quantum-inspired stochastic dynamics and used ablation to isolate the contribution of the dynamic component. Outside language, Yao et al. (2026) combined a quantum-inspired embedding with physics-informed neural prediction and multi-source data fusion. These systems are not models of literary cognition, but they illustrate three practices that QSD adopts: modular architecture, frozen or explicit interfaces between modules, and comparative tests that ask whether the quantum-inspired component adds predictive value beyond classical alternatives.

Classical theories already explain many phenomena that matter for reading. Construction-integration models describe the interaction of textual input and background knowledge (Kintsch, 1988), and situation models explain how readers maintain and revise representations of events across discourse (Zwaan and Radvansky, 1998). Bayesian, hidden-state, and classical state-space models can also produce sequential revision when the latent state is allowed to change over time. QSD therefore has no empirical advantage merely because literary meaning is ambiguous, dynamic, multiscale, or order sensitive. Its burden is stronger: a common state and probe geometry must be fixed independently of the confirmatory response table; the resulting quantum-probabilistic submodel must satisfy its own structural constraints; and any predictive advantage must be evaluated against explicitly specified classical comparators rather than against an undefined notion of “classical probability.”

### 2.3 Model positioning and claim boundary

Three logically distinct claims are separated throughout this paper. First, “quantum-inspired” is representational: Hilbert spaces, density operators, projectors, or channel notation may provide a compact model without implying anything intrinsically quantum about the data. Second, “quantum-probabilistic” denotes a constrained model in which a shared state and frozen probe geometry are tied to Born probabilities, the restricted contextual channel specified below, and, for the A/B diagnostic, projective Lüders updating and the QQ equality. This second level is the claim advanced by QSD. Third, “intrinsic nonclassicality” is reserved for observed statistics shown incompatible with an explicitly defined classical representability criterion. Context effects, fitted noncommuting operators, or agreement with the QQ equality do not by themselves establish that stronger claim. A dedicated contextuality analysis would be required to separate direct contextual influences from failure of a classical coupling or joint representation (Dzhafarov and Kujala, 2016).

Accordingly, “classical” in the empirical comparisons below always refers to a stated comparator class, not to every conceivable latent-variable or context-indexed stochastic construction. QSD can earn support relative to preregistered classical alternatives while leaving open the logically stronger question of whether a sufficiently flexible classical contextual model could reproduce the same finite response table.

## 3 QSD model architecture

### 3.1 Problem definition

At a preregistered narrative checkpoint, QSD represents the population distribution of interpretive dispositions before focal probing and models how that state is read out and updated as later context arrives. The confirmatory task has four linked requirements: define a local semantic state and independently calibrated probe geometry; identify that state from observed response marginals; restrict contextual evolution so that it cannot absorb arbitrary response patterns; and evaluate sequential and predictive behavior against explicit classical comparators. Probe geometry and context-target rules are fixed outside the held-out confirmatory sample.

### 3.2 Overall framework

Figure 1 summarizes the QSD pipeline. Pilot calibration fixes the A/B/C probe geometry and the context-target rule. In confirmation, the A → B, B → A, and C-only arms identify the population state; the constrained contextual channel updates that state after new narrative input; and the A/B sequence is evaluated by the QQ and incompatibility constraints. Section 5 then compares QSD with preregistered classical model classes under held-out evaluation. Separating calibration, identification, dynamics, and adjudication prevents any single module from being refitted solely to rescue the confirmatory response table.

**Figure 1:**
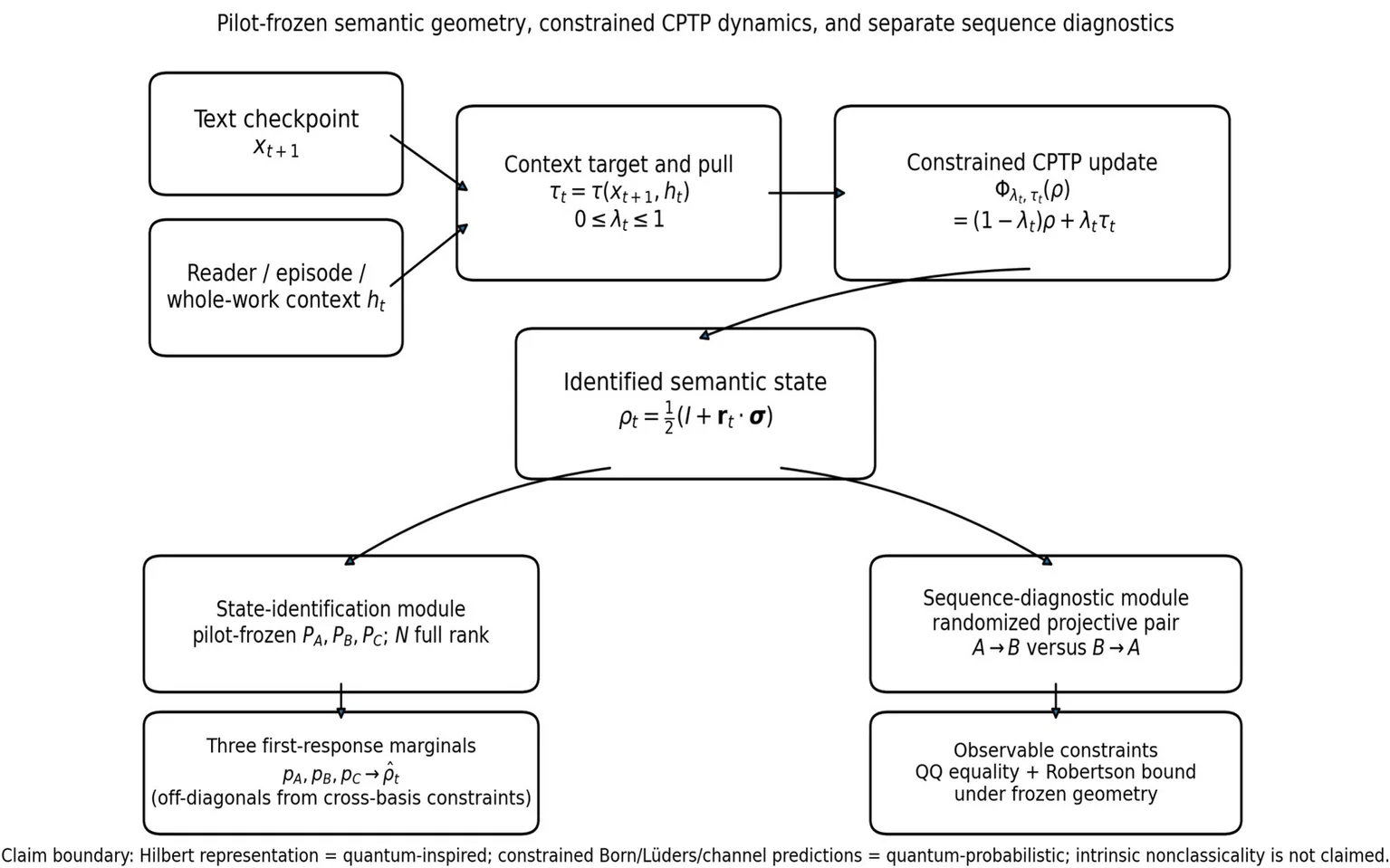
QSD constrained quantum-probabilistic architecture. Pilot-frozen probe geometry identifies the population state, while a restricted CPTP channel updates that state toward a preregistered context target. The A/B sequence branch supplies model-specific QQ and incompatibility constraints; none of these representational choices alone is treated as evidence of intrinsic nonclassicality.

### 3.3 Module I: probe calibration and semantic-state identification

The first confirmatory implementation fixes a local two-dimensional complex Hilbert space at a preregistered narrative checkpoint. The choice *d* = 2 is a parsimonious model restriction for one local interpretive contrast, not an ontological claim about literary meaning. It is the smallest Hilbert space that supports distinct, potentially noncommuting binary probe bases. The state is population-level: it summarizes interpretive dispositions in a randomized reader sample before any focal probe is asked. In a convenient Bloch representation, the checkpoint state is


$$\rho_{t}=½(I+r_{t}\cdot \sigma ),\|r_{t}\|\leq 1$$ (1)

where r_t_ is a three-component Bloch vector and σ denotes the Pauli operator basis used only as a compact parameterization of a 2 × 2 positive, unit-trace matrix. For a probe j with unit Bloch direction n_j_, the yes-projector is P_j_^+^ = ½(I + n_j_·σ), its complement is P_j_^−^ = I − P_j_^+^, and the first-response probability is


$$p_{j}=\mathrm{Tr}(\rho_{t}\;P_{j}^{+})=½(1+r_{t}\cdot n_{j})$$ (2)

Equation 2 gives the yes-response probability for a probe direction fixed in the pilot. For the focal fish interpretation in The Old Man and the Sea, pilot work calibrates three questions at the same checkpoint. Probe A asks whether the fish is being construed primarily as quarry or biological object; probe B asks whether it is being construed as a relational counterpart; probe C asks whether it is being construed as an existential mirror of Santiago’s struggle. These labels are not three mutually exclusive classical categories. Each binary question defines a different projection of the same local state. Probe geometry is estimated only in an independent pilot sample and is frozen before confirmatory data are examined. A convenient gauge sets n_A_ = (0,0,1), n_B_ = (sin θ_B_, 0, cos θ_B_), and n_C_ = (sin θ_C_ cos φ_C_, sin θ_C_ sin φ_C_, cos θ_C_), with θ_B_, θ_C_, and φ_C_ calibrated in the pilot. The overlap Tr(Pᵢ^+^P_j_^+^) = ½(1 + nᵢ·n_j_) fixes the inner-product geometry used in confirmation; it is not refitted to improve the confirmatory response table.

One binary probe pair is not sufficient to identify a general 2 × 2 density state. The state in Equation 1 has three independent real parameters. After the probe directions are fixed in a separate pilot sample, three linearly independent binary bases supply three independent expectation constraints. Let N be the 3 × 3 matrix whose rows are n_A_ᵀ, n_B_ᵀ, and n_C_ᵀ, and let p = (p_A_, p_B_, p_C_)ᵀ. Then


$$\mathrm{2p}-1=N\;r_{t},\mathrm{so}\;\text{that}\;r_{t}=N^{-1}(\mathrm{2p}-1)\;\text{when}\;\det(N)\neq 0$$ (3)

Thus the minimal *d* = 2 design requires three preregistered, linearly independent binary probe bases. A and B are the two ordered probes used for the QQ diagnostic; C is an independent third basis used only for state identification. In the confirmatory sample, p_A_ is estimated from the first response in the A → B arm, p_B_ from the first response in the B → A arm, and p_C_ from a C-only arm. Under finite-sample noise, direct inversion is replaced by constrained maximum-likelihood estimation over ||r_t_|| ≤ 1. The rank and condition number of N are checked in pilot calibration and frozen before confirmation. More generally, a d-dimensional density operator has *d*^2^–1 independent real parameters, so higher-dimensional QSD would require an informationally complete measurement design; that extension is outside the confirmatory scope of the present paper.

Choose the A probe as the reference basis. Then ρ_t_ can be written as [[(1 + r_z_)/2, (r_x_ − ir_y_)/2], [(r_x_ + ir_y_)/2, (1 − r_z_)/2]]. The off-diagonal entry is not assumed to represent a physical coherence process. Operationally, it contains the components of the reconstructed state that are invisible to the A marginal but are required to reproduce judgments in the independently calibrated B and C bases. In the special calibration gauge n_B_ = (1,0,0) and n_C_ = (0,1,0), Re(ρ₁₂) = p_B_ − ½ and Im(ρ₁₂) = −(p_C_ − ½); for general frozen geometry the same quantities follow from Equation 3 through N^−1^. If B and C do not supply stable independent constraints, the off-diagonal terms are not identified and the density-state representation fails its own admission criterion (see Table 1).

**Table 1:** Components of the minimal *d* = 2 QSD identification module.

Component	Specification	Role
State ρ_t_	Population-level 2 × 2 density state	Represents the semantic state at the focal checkpoint
Probe A	Pilot-frozen binary basis P_A_±	First response in A → B; one QQ probe
Probe B	Independent pilot-frozen basis P_B_±	First response in B → A; second QQ probe
Probe C	Third independent binary basis P_C_±	Completes state identification in a C-only arm
Estimator	Constrained MLE with ||rˆ_t_|| ≤ 1	Reconstructs a valid state from A/B/C marginals

### 3.4 Module II: constrained contextual dynamics

Contextual dynamics are deliberately restricted rather than left as an arbitrary latent transition. New text and validated higher-scale context determine a preregistered target density state τ_t_ = τ(x_t+1_,h_t_), while a bounded parameter λ_t_ controls the strength of contextual pull:


$$\rho_{t+1}=\Phi \lambda_{t},\tau_{t}(\rho_{t})=(1-\lambda_{t})\rho_{t}+\lambda_{t}\tau_{t},0\leq \lambda_{t}\leq 1$$ (4)

Here x_t+1_ is the newly encountered passage and h_t_ contains preregistered reader, episode, and whole-work context. The target τ_t_ is fixed by an independent calibration rule for that textual/contextual condition; λ_t_ is either preregistered or estimated on training data and frozen before held-out evaluation. Equation 4 is a completely positive trace-preserving (CPTP) channel by construction. If τ_t_ = Σ_a_ t_a_|a⟩⟨a| and {|j⟩} is any orthonormal basis, one Kraus representation is K₀ = √(1 − λ_t_)I and K_aj_ = √(λ_t_t_a_)|a⟩⟨j|, for which ΣK†K=I and ΣKρK† = (1 − λ_t_)ρ + λ_t_τ_t_. The update therefore contracts the current semantic state toward a context-specified target rather than allowing an unrestricted map to absorb arbitrary response patterns. This CPTP restriction disciplines the dynamics; it is not itself claimed as evidence of intrinsic nonclassicality.

## 4 Sequential diagnostic and complete QQ derivation

### 4.1 Sequential probabilities for two literary probes

Let P=P_A_^+^ and Q = P_B_^+^ denote the yes-projectors for the two focal literary probes, with complements I − P and I − Q. The QQ diagnostic uses the same pre-probe population state ρ_t_ in two randomized groups. Under projective measurement with Lüders updating, the joint probability of yes to A followed by no to B is


$$p(\text{AyBn})=\mathrm{Tr}[(I-Q)P\rho_{t}P(I-Q)]=\mathrm{Tr}[\rho_{t}\;P(I-Q)P]$$ (5)

Equation 5 gives the A_y_B_n_ sequential probability; the other three cross-answer probabilities are obtained by replacing P and Q with their complements in the same way. This sequence model is narrower than the general contextual update in Equation 4: between A and B, no new narrative input or additional transition is permitted.

### 4.2 Derivation of the QQ equality

Define the QQ statistic as


qQQ=p(AyBn)+p(AnBy)−p(ByAn)−p(BnAy) (6)

Using trace cyclicity and the projective identities P^2^ = P and Q^2^ = Q, Equation 6 can be written qQQ = Tr(ρ_t_K), where


K=P(I−Q)P+(I−P)Q(I−P)−Q(I−P)Q−(I−Q)P(I−Q) (7)

Equation 7 defines the operator K entering the QQ statistic.

Equation 8 gives the expansion of the first two terms.


P(I−Q)P+(I−P)Q(I−P)=P+Q−PQ−QP (8)

Equation 9 shows that the last two terms have the same expansion:


Q(I−P)Q+(I−Q)P(I−Q)=P+Q−PQ−QP (9)

Therefore, K = 0 and, for any initial density state and any Hilbert-space dimension satisfying the assumptions below,


$$\mathrm{qQQ}=\mathrm{Tr}(\rho_{t}K)=0$$ (10)

This is the parameter-free QQ equality derived by Wang and Busemeyer (2013). In the present paper, satisfying Equation 10 supports compatibility with the binary projective Lüders submodel; it does not demonstrate that the response statistics lack a classical contextual or sequential representation. Conversely, a clear violation rejects this specific projective implementation, not every quantum-probabilistic model or generalized measurement instrument. Within this projective submodel, noncommutativity is necessary for a genuine order effect, but neither fitted noncommutativity nor QQ compatibility establishes intrinsic nonclassicality. The QQ equality constrains the pattern of the four cross-answer probabilities and is adjudicated together with explicit classical comparators.

### 4.3 Assumptions required in a literary application

The algebraic identity becomes an empirical prediction only when the literary experiment implements the same measurement assumptions. Table 2 makes those assumptions explicit. In particular, the two questions must concern the same pre-probe narrative state, no new text or feedback may intervene between them, and the first answer must be the only state-changing event before the second question. Probe C is therefore kept outside the A/B sequence.

**Table 2:** QQ assumptions and failure conditions in the literary experiment.

Assumption	Experimental requirement	Failure condition
Binary exhaustive projectors	Two exhaustive outcomes for each focal probe	Non-exhaustive or overlapping categories
Lüders sequential update	First response induces only the projective update	Additional response-dependent transformation
Common pre-sequence state	Random assignment to A → B and B → A at the same checkpoint	Systematic pre-probe group differences
Stable probe operators	Same wording, coding, and geometry across orders	Probe definition changes with order
No intervening input	No new text, feedback, explanation, or delay	Context changes between the two probes
Same narrative checkpoint	A and B refer to the same passage state	Different checkpoints or texts
Complete joint-response data	Retain all four outcomes under preregistered exclusions	Selective missingness or exclusion
Separate C arm	C is measured independently of the A/B sequence	C is inserted into the sequence or reused *post hoc*

### 4.4 Operational consequence of probe incompatibility

The frozen A/B geometry also yields an internal operational constraint beyond the formal statement [P_A_, P_B_] ≠ 0. For a binary projector P with yes-probability p, ΔP = √[p(1 − p)]. The Robertson relation therefore gives


$$\Delta P_{A}\;\Delta P_{B}\geq ½\;|\mathrm{Tr}(\rho_{t}[P_{A},P_{B}])|$$ (11)

For qubit projectors P_A_ = ½(I + n_A_·σ) and P_B_ = ½(I + n_B_·σ), Equation 11 becomes:


$$\surd [p_{A}(1-p_{A})p_{B}(1-p_{B})]\geq ¼\;|(n_{A}\times n_{B})\cdot r_{t}|$$ (12)

Every term on the right side is fixed or identified by the pilot-frozen geometry and the three-basis state reconstruction, so Equation 12 is empirically checkable without introducing a new fitted operator. It is an internal consistency condition of the chosen Hilbert-space model (Robertson, 1929), not a nonclassicality witness: its lower bound can be zero for some states even when the projectors do not commute, and classical contextual constructions may reproduce the same marginal and sequential statistics.

## 5 Prospective empirical adjudication

### 5.1 Pilot calibration

Pilot work has five jobs: select a focal checkpoint, establish interpretable A/B/C probe wording, estimate the frozen projector geometry, define the context-to-target rule τ(x,h) used by Equation 4, and verify that N is sufficiently far from singular to support stable state reconstruction. Probe geometry and the τ rule are estimated only in the pilot phase. A convenient gauge sets n_A_ along one coordinate axis and places n_B_ in a reference plane; n_C_ must contribute an independent third direction. The exact calibration loss, rank and conditioning thresholds, wording rules, target-state construction, and exclusion criteria are preregistered and then frozen. This separation prevents the confirmatory sample from being used both to invent the semantic geometry or dynamics and to test them.

### 5.2 Confirmatory sample and state reconstruction

At the same narrative checkpoint, readers are randomly assigned to three arms: A → B, B → A, or C-only. The first response in each arm estimates one of p_A_, p_B_, or p_C_ before any focal probe has altered the state. Those three marginals reconstruct the population-level ρ_t_ through Equation 3, using constrained estimation when sampling noise would otherwise place rˆ_t_ outside the Bloch ball. The full two-question response tables from the A → B and B → A arms are reserved for the QQ test and are not used to redefine the probe geometry.

### 5.3 Classical comparators and decision rules

The classical comparison is defined by model class rather than by a generic label Figure 2. C0 is a commutative static model with one order-independent joint distribution and no measurement-induced state change. C1 is a matched classical sequential latent-state model—such as a finite hidden-state or state-space process—that is allowed to update after the first response and can therefore produce history dependence and question-order effects. Its state dimension, regularization, and effective parameter budget are matched as closely as possible to QSD. C2 denotes a broader contextual classical benchmark in which variables or transitions may be explicitly indexed by measurement context. Such a model can be highly flexible and may reproduce response tables that also admit a Hilbert-space representation. QSD is not claimed to exclude every member of C2. Instead, the confirmatory claim is comparative: QSD must satisfy its own quantum-probabilistic constraints and achieve held-out performance at least as good as the preregistered C0/C1 comparators after complexity control. Outperforming a particular C0 or C1 comparator would establish comparative adequacy, not a universal quantum/classical separation. If a scientifically meaningful C2 model achieves equal predictive adequacy without the QSD structure, no empirical exclusivity is claimed. A stronger statement of intrinsic contextuality would require a separate design and classical representability criterion, for example a context–content analysis in the sense of Contextuality-by-Default (Dzhafarov and Kujala, 2016).

**Figure 2:**
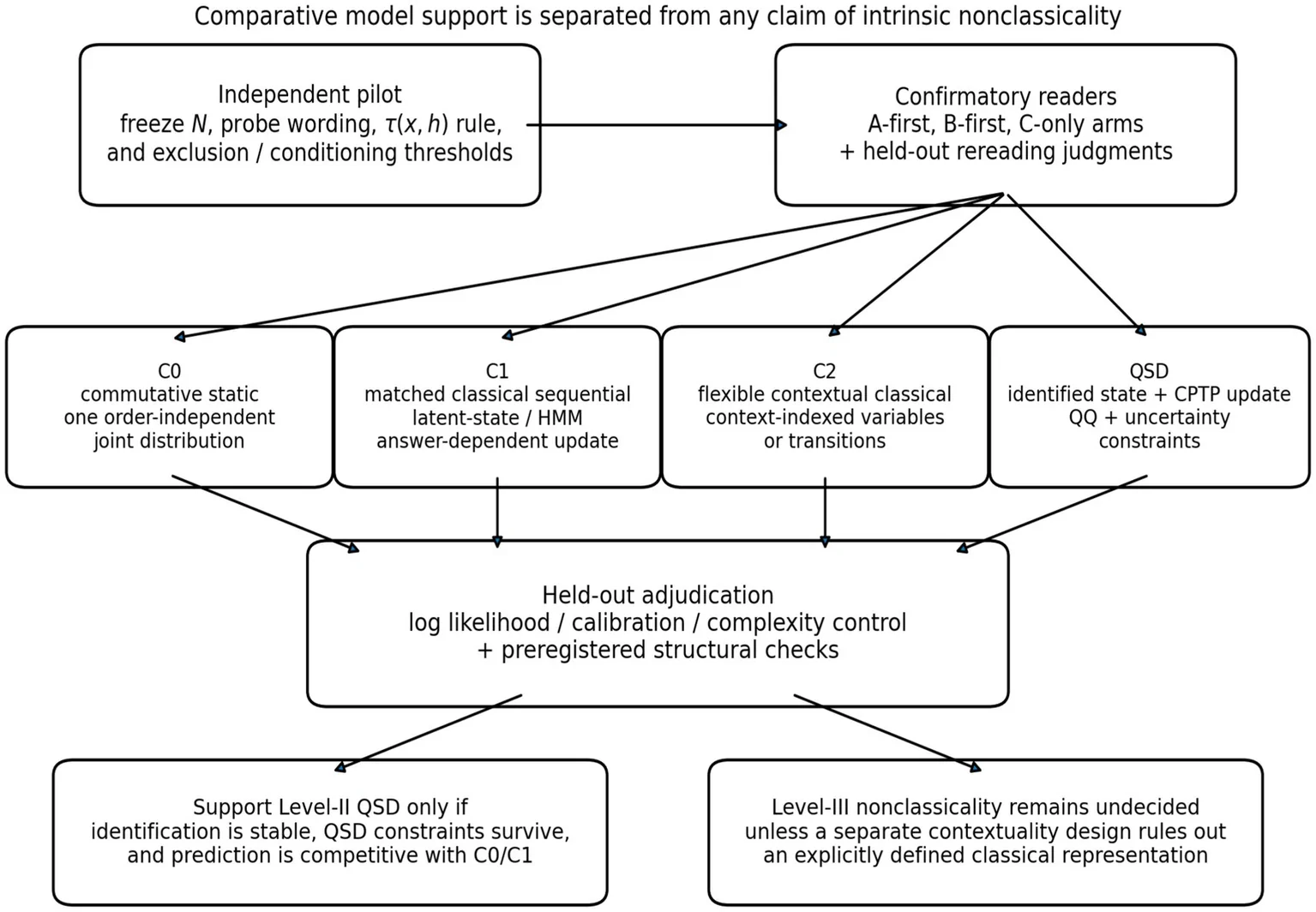
Empirical adjudication across quantum-probabilistic and classical model classes. Pilot calibration freezes probe geometry and the context-target rule before confirmation. The three-arm design reconstructs the state, the A/B sequence tests QQ and incompatibility consistency, and held-out prediction is compared with C0/C1 and, where feasible, a broader contextual classical benchmark C2. Passing these tests supports the QSD model class but is not labeled proof of intrinsic nonclassicality.

### 5.4 Three empirical commitments

P1—Identifiable state and quantum-probabilistic sequence structure. The three-basis design must reconstruct a stable population state under frozen geometry; the A/B projectors must satisfy the uncertainty consistency condition; and the randomized A → B/B → A response tables must be compatible with the preregistered QQ equality. Failure of rank, unstable reconstruction, violation of an internal consistency constraint, or a clear QQ violation rejects this projective implementation. If no stable order structure is observed, the sequence component loses its empirical motivation even if the QQ equality is satisfied trivially. Passing these tests supports Level-II quantum-probabilistic compatibility, not Level-III intrinsic nonclassicality.

P2—Contextual reopening. After a passage temporarily concentrates endorsement on one interpretation, a later preregistered narrative cue should restore measurable support to an alternative interpretation. This dynamic claim loses support when a matched classical sequential model predicts the reopening trajectory equally well or better.

P3—Multiscale predictive gain. Delayed judgments about an earlier passage should be predicted better when validated episode- or whole-work context is available to the constrained channel in Equation 4 than when the same model is restricted to local history. If higher-scale inputs provide no held-out gain, the multiscale component is removed.

## 6 Discussion

### 6.1 From a general formalism to an identifiable literary model

The central methodological point is formal economy rather than expansion of quantum vocabulary. A Hilbert-space representation is Level I: it can be useful without being uniquely quantum. QSD advances the narrower Level-II claim that one shared state, frozen probe geometry, Born probabilities, a constrained CPTP contextual channel, and a projective Lüders sequence jointly impose testable structure. Level III—intrinsic nonclassicality of the observed statistics—is not claimed here. A density state is retained only because three independent probe bases can identify it; the off-diagonal entries are retained only insofar as they are fixed by cross-basis judgments; and the QQ pair is retained only because it yields a parameter-free equality with explicit assumptions. This separates representation, quantum-probabilistic constraint, and nonclassicality rather than treating them as interchangeable.

The recent quantum-inspired architectures cited above reinforce this modular discipline. Their domains differ from literary cognition, yet their strongest methodological lesson is architectural rather than terminological: make state representation, transformation, measurement, and comparison modules visible; freeze interfaces where possible; and use ablation or matched baselines to determine whether a quantum-inspired component adds predictive value. QSD applies that discipline to reader judgments while keeping the stronger classical/nonclassical question separate. In particular, fitted noncommutativity is treated as a property of the model geometry; only observable constraints generated by that geometry enter adjudication.

### 6.2 Causal-logical prospect

The present model addresses probabilistic identification and sequential judgment. Equilibrium-based and causal-logical approaches to quantum intelligence propose broader formalisms involving dynamic balance, logically defined causality, and, in some formulations, background-independent relations among information-processing systems (Zhang, 2023, 2024; Namazi et al., 2026). Those proposals identify a possible future layer rather than a missing component of the present confirmatory test. Any causal-logical extension would need to add an independently testable restriction beyond the CPTP channel in Equation 4 or to the relation among semantic states; otherwise it would enlarge the vocabulary without changing adjudication.

### 6.3 Limitations and expected failure modes

The current implementation deliberately limits identification to a two-dimensional local semantic subspace and reconstructs a population-level state rather than an individual reader’s state. The *d* = 2 choice and probe geometry are model assumptions calibrated in an independent pilot, not discoveries forced by the confirmatory sample. Three linearly independent bases are minimally sufficient in *d* = 2, but near-collinearity can make reconstruction unstable even when det N is nonzero; a preregistered conditioning threshold is therefore required. The constrained channel in Equation 4 is CPTP but intentionally simple; failure of its context-target contraction should motivate rejection or replacement rather than post-hoc expansion. The QQ equality depends on binary projectors, Lüders updating, stable wording, and the absence of intervening narrative input. The Robertson bound supplies an internal compatibility check but is not a contextuality witness. Finally, no empirical data are reported here, and the proposed tests do not purport to exclude every flexible classical contextual model. A Level-III nonclassicality claim would require a separate contextuality design and explicit classical representability criterion.

## 7 Conclusion

Quantum Semantic Dynamics is formulated here as an identifiable and constrained quantum-probabilistic literary model. At each focal checkpoint, pilot-frozen probe geometry and three independent binary bases identify a low-dimensional population state, including the cross-basis components represented by its off-diagonal entries. New textual and higher-scale context act through a preregistered CPTP contraction rather than an arbitrary transition. Two probes form a randomized projective sequence with a fully derived QQ constraint and an operational uncertainty consistency relation. The model earns support only if identification is stable, its own structural constraints survive, and held-out prediction is competitive with explicitly defined classical alternatives. Such evidence would support QSD as a quantum-probabilistic model class; it would not by itself establish that literary cognition is intrinsically nonclassical. This boundary makes the proposal more, not less, testable: it states exactly what future data can support, reject, or leave undecided.
